# Plasma-derived exosomal miR-4732-5p is a promising noninvasive diagnostic biomarker for epithelial ovarian cancer

**DOI:** 10.1186/s13048-021-00814-z

**Published:** 2021-04-28

**Authors:** Jingjing Liu, Jigeun Yoo, Jung Yoon Ho, Yuyeon Jung, Sanha Lee, Soo Young Hur, Youn Jin Choi

**Affiliations:** 1 Department of Obstetrics and Gynecology, Seoul St. Mary’s Hospital, College of Medicine, The Catholic University of Korea, Seoul, Republic of Korea; 2 Cancer Research Institute, College of Medicine, The Catholic University of Korea, Seoul, Republic of Korea; 3 Department of Obstetrics and Gynecology, Affiliated Hangzhou First People’s Hospital, Zhejiang University School of Medicine, Hangzhou, China

**Keywords:** Exosomes, Exosomal miRNA profiling, microRNAs, Epithelial ovarian cancer, miR-4732-5p

## Abstract

**Background:**

Exosomal miRNAs regulate gene expression and play important roles in several diseases. We used exosomal miRNA profiling to investigate diagnostic biomarkers of epithelial ovarian cancer (EOC).

**Methods:**

In total, 55 individuals were enrolled, comprising healthy (*n* = 21) and EOC subjects (*n* = 34). Small mRNA (smRNA) sequencing and real-time PCR (RT-PCR) were performed to identify potential biomarkers. Receiver operating characteristic (ROC) curves were conducted to determine biomarker sensitivity and specificity.

**Results:**

Using smRNA sequencing, we identified seven up-regulated (miR-4732-5p, miR-877-5p, miR-574-3p, let-7a-5p, let-7b-5p, let-7c-5p, and let-7f-5p) and two down-regulated miRNAs (miR-1273f and miR-342-3p) in EOC patients when compared with healthy subjects. Of these, miR-4732-5p and miR-1273f were the most up-regulated and down-regulated respectively, therefore they were selected for RT-PCR analysis. Plasma derived exosomal miR-4732-5p had an area under the ROC curve of 0.889, with 85.7% sensitivity and 82.4% specificity in distinguishing EOC patients from healthy subjects (p<0.0001) and could be a potential biomarker for monitoring the EOC progression from early stage to late stage (*p* = 0.018).

**Conclusions:**

Plasma derived exosomal miR-4732-5p may be a promising candidate biomarker for diagnosing EOC.

**Supplementary Information:**

The online version contains supplementary material available at 10.1186/s13048-021-00814-z.

## Introduction

Epithelial ovarian cancer (EOC) which comprises the majority of malignant ovarian tumors ranks the third most common and the most lethal reproductive cancer in women with 22,240 new cases and 14,070 deaths occurred worldwide in 2018 [1]. Cancer prevention and early detection is a research priority, however due to inefficient and insensitive diagnostics such as transvaginal ultrasound (TVU) and 125 (CA 125) blood tests, success rates are varied [2, 3]. The absence of typical symptoms for most of cases led the most of the ovarian cancer patients be diagnosed at late stage (III-IV), yielding a low 5-year relative survival rate of 43% [1]. Based on current methodology, several molecules including DNA [4], proteins [5], and mRNA and miRNAs [6] have shown great potential as biomarkers for the detection of ovarian cancer and its invasive development.

Exosomes are vesicles secreted by cells, with a diameter ranging between 30 nm–100 nm. They carry functionally informative molecules which transfer information across plasma membranes. Exosomes are naturally detected in biological fluids such as blood [7], urine [8], ascites [9], and amniotic fluid [10]. Almost all cell types, including tumors, immune, nerves, and stem cells produce and release exosomes. The molecules directly activate receptors through cell membrane receptors, and also transport proteins, messenger RNA, miRNA, long non-coding RNA, circular RNA, lipids, DNA, and even organelle into receptor cells to participate in intercellular communications [11–14]. Exosomes play key roles in physiological and pathological processes, such as immune and inflammatory responses, angiogenesis, apoptosis, coagulation, waste disposal. It may be used as early diagnostic biomarkers for a variety of diseases and also can function as drug carrier molecules to specific targets in certain areas and tumors [15–18]. A paradigm of miRNAs entrapped in exosomes in blood could act as hormones, then enter the circulating environment and travel to distant organs, leading to widespread consequences within the recipient cells at a distance from the donor cells in both autocrine and paracrine signaling pathway [19]. Tumor microenvironmental interactions were also mediated by miRNA biogenesis, methylation, and transcriptional changes where these miRNAs were secreted through micro-vesicles or exosomes to direct cell-to-cell signaling and promote cancer cell proliferation and metastasis [20]. Thus, exosomes appear to be a novel and significant signaling metastatic factor in the tumor microenvironment. It can induce immunosuppression and immune escape [21]. In addition, exosome-facilitated drug could be loaded by chemotherapeutics, miRNAs and siRNAs to treat certain disease [21].

Recently, several studies have indicated that abundant miRNAs and other small RNAs (smRNAs) are present in biofluids, e.g. serum [22], plasma [23], urine [24], and cerebral spinal fluid [25]. MicroRNAs can stably exist in body fluids in extracellular vesicles (EVs), including exosomes, or bound to proteins or lipids [26], and play crucial roles in intercellular communications [27]. Importantly, disease-associated changes in biofluid miRNA expression profiles may serve as bio-indicators of pathological status, and as such, are promising biomarkers for several cancers [28, 29]. For example, the combination of plasma derived miR-21, miR-145, and miR-155 were used to early detect lung cancer from healthy smokers with 69.4% sensitivity and 78.3% specificity [30]. High elevated expression of miR-92 could be a potential marker for colorectal cancer screening with a high sensitivity of 89% and specificity of 70% [31]. Over-expressed miR-221 and let-7a predicted a favorable outcome of treatment for non-small-cell lung carcinoma, as opposed to elevated miR-137, miR-372, and miR-182 with poor prognosis [32]. Importantly, specific biomarkers could monitor the effectiveness of experimental therapies during clinical trials (e.g. NCT01631760 and NCT3713320). What’s more, in current preclinical development, mimicing tumor suppressive miRNAs or suppressing onco-miRs (using anti-miRs) are the strategies to modulate therapeutic approaches to cancer [33]. For example, in a phase I clinical trial (NCT01829971), miR-34 mimics, encapsulated in lipid nano-particles (MRX34) was treated with primary liver cancer or other selected solid tumors or hematologic malignancies with an acceptable safety and antitumor activity in a subset of patients with refractory advanced solid tumors [34]. An extension to a number of diseases indications beyond cancer was also investigated in mouse models of hepatitis, cardiac diseases and diabetes associated kidney fibrosis [33]. miRNAs were also related to prognosis, diagnosis, and chemotherapy sensitivity and could play a role of monitoring the treatment response and relapse in ovarian cancer [20, 35]. A systematic review including 497 articles summarized that upregulation of miR-149, miR-155, miR152, miR-199a, miR200b, miR200c, miR-30d, miR-34c, miR-363, miR-497, miR-506, miR-9, and let-7i, and downregulation of miR-23a and miR-603 could prohibit platinum resistance [20]. In epithelial ovarian cancer, the miR-200 family was reported as the most significantly upregulated miRNAs in Iorio’s study, showing potential ability as diagnostic biomarkers [36]. Serum exosomal miR-1307 and miR-375 could enhance the diagnostic accuracy of traditional biomarkers when combined with CA-125 and HE4 in ovarian cancer [37].

A variety of techniques can be applied to conduct miRNA profiling. For example, quantitative reverse-transcription polymerase chain reaction (qRT-PCR) assays, Northern blotting analyses, and microarrays have been used extensively in miRNA profiling studies [38–40]. Recently, the newly developed technology, next-generation sequencing (NGS) has obtained much attention in smRNA profiling due to its unique advantages in terms of test specificity, sensitivity, and robust smRNA identification [41, 42].

In this study, we sought to elucidate potential miRNA markers for EOC using smRNA sequencing. Six plasma samples from EOC patients and four healthy controls were selected and analyzed using smRNA sequencing to identify correlations between differential miRNA expression levels in EOC. RT-qPCR was used to validate promising deregulated miRNA candidates in another dataset including 17 healthy subjects and 28 ovarian cancer subjects to identify novel noninvasive diagnostic biomarkers for ovarian cancer.

## Materials and methods

### Patients and plasma samples

Between September 2009 and October 2015, peripheral whole blood was collected in anticoagulant tubes from healthy subjects (*n* = 21) at Seoul St. Mary’s Hospital Biobank, and papillary serous carcinoma ovarian cancer patients (*n* = 34) before initial treatment at the Department of Obstetrics and Gynecology, Seoul St. Mary’s Hospital. Blood collection and associated studies were performed with approval from the ethics committee of the Catholic University of Korea, College of Medicine (IRB approval, KC17TESI0690) in compliance with the Helsinki Declaration. Written informed consent was obtained from every participant before sample collection.

### EV preparation

Blood samples were centrifuged at 2000×g for 10 min, and plasma EV RNA isolation was performed based on a polyethylene glycol (PEG)/dextran (DEX) aqueous two-phase systems (ATPSs) [43]. Briefly, 200 μl plasma was mixed with 40 μl ATPSs solution, prepared with PEG/DEX (Sigma-Aldrich, St. Louis, MO, USA) and dissolved in phosphate buffered saline (PBS) at 21%/9% (wt/wt) concentration. The upper PEG phase was separated and removed after centrifugation at 3000×g for 30 min at 4 °C, while the pellet/DEX phase was resuspended in PBS for further EV analysis.

### Nanoparticle tracking analysis (NTA)

NTA method is thought as a gold standard to measure the concentration of exosomes and shed microvesicles [44]. Each sample was diluted in 200 μl filtered PBS to generate 1/1000–1/50000 dilutions. EV size and numbers in the DEX phase were analyzed by NTA (ExoCope monoTM, Exosome Plus, Inc. South Korea). Recorded frame number per file was > 144, and measurements were repeated 15 times in different sub-volume positions at 25 °C. Laser exposure times were 2 ms and the camera gain was 30. Analyses were performed with ExoCope tracker software, version 1.005 (ExoCope monoTM). The intensity threshold was set to 20 (quadrature noise level in digital number per pixel); minimum tracked particle size was set to 50 nm; minimum separable particle distance was set to 5.7 pixels. The system was calibrated using 100 nm polystyrene beads purchased from Thermo Scientific (Fremont, USA) at five different concentrations.

### Transmission electron microscopy (TEM)

To verify intact EVs, TEM was performed as previously described [44, 45]. Briefly, EVs were fixed in 4% paraformaldehyde and 2% glutaraldehyde in PBS (pH 7.4). Samples were deposited onto formvar carbon-coated, glow discharged grids (FCF300-cu, Electron Microscopy Science, Hatfield, PA, USA), stained with 7 μl 2% uranylacetate and embedded in methylcellulose/uranylacetate for 10 s. The grids were air-dried for 30 min, then imaged on TEM (JEM-1011, Japan) at a 60-kV acceleration voltage.

### SmRNA library preparation and sequencing

To extract exosomal miRNAs from plasma, total exosomal miRNAs were purified using Qiagen’s miRCURY Exosome serum/plasma kit according to the protocol (Qiagen, CA, USA). RNA concentrations were measured using a NanoDrop instrument (Thermo Scientific) and samples were sent to Macrogen (Seoul, Korea) for smRNA sequencing. Briefly, total RNA quality (1 μg) with poly (A) mRNA enriched and magnetic beads with an oligo (dT) primer were assessed as the input before cDNA library construction and then generated using a SMARTer smRNA-Seq kit (Takara Bio, Shiga, Japan) as previously described [46]. Purified mRNAs were disrupted into short fragments and double-stranded cDNAs were immediately synthesized. Library preparation was performed as previously described [47]. Details are provided in the Supplementary methods and materials File.

### Quality control of smRNA sequencing data

Uniquely clustered reads were sequentially aligned to the reference genome (hg38), the non-coding RNA database, miRBase v21 miRBase database [48] (http://www.mirbase.org/), and RNAcentral 10.0 (https://rnacentral.org/) to identify known miRNAs and other RNA types such as transfer RNAs (tRNAs), Small nuclear RNA (snRNAs), small nucleolar RNAs (snoRNAs) etc. Data quality was determined by phred quality scores at each cycle, with a created FastQC file (http://www.bioinformatics.babraham.ac.uk/projects/fastqc). Unmatched trimmed reads to non-miRNAs in the Rfam database (http://rfam.xfam.org/) were considered potential novel miRNAs.

### RNA isolation, reverse transcription and qRT-PCR

Plasma based miRNA expression was measured using the miRCURY exosome serum/plasma kit (Qiagen). Total RNA was reverse-transcribed to cDNA using the HB miR RT KitTM System I (Heimbiotek, Seongnam, Korea). miRNA sequence (5′-3′) for hsa-miR-1273f and hsa-miR-4732-5p were UGUAGAGCAGGGAGCAGGAAGCU and GGAGAUGGAGGUUGCAGUG, repectively. RT and PCR primers were designed and synthesized by Heimbiotek company (Seongnam, Korea). We used 20 μl working volumes for RT-PCR reactions using the SYBER green HB miR RT-PCR master mix KitTM System II (Heimbiotek) following manufacturer’s protocols.

RT-PCR was conducted at 16 °C for 30 min, 42 °C for 30 min, and 85 °C for 5 min on a MyGenie 96 Thermal Block (Bioneer, Daejeon, Korea). Quantitative RT-PCR was performed at 95 °C for 15 min, and 45 cycles at 95 °C for 30 s, 60 °C for 40 s, and 72 °C for 30s a on Viia7 Real-Time PCR system (Thermo Fisher Scientific). Each qRT-PCR assay was performed in triplicate. Quant-StudioTM RT-PCR software (v1.1) was used to determine quantification cycle. Ct was defined as the fractional cycle number at which fluorescence exceeded a given threshold. *Caenorhabditis elegans* miR cel-miR-39 (cel-miR-39) was used as a stable reference control (Heimbiotek, Seongnam, Korea). Relative quantification was performed using the 2-dCt method (2-ΔΔCt), where dCt = Ct [miRNA] - Ct [cel-miR-39].

### Statistical analysis

Hierarchical clustering (heat-maps) and volcano plots were performed using R. Differentially expressed miRNAs (DEmiRs) were analyzed by edgeR with a cutoff of |Log_2_fold change | ≥ 1 and *p*-value ≤0.05. Statistical analyses were performed using GraphPad Prism software Ver. 8 (GraphPad Inc., San Diego, CA, USA). All data were presented as the mean ± standard deviation (SD), or the median depending on data distribution. Two tailed Student’s t-test or Mann–Whitney U-tests were used to analyze differences in exosomal miRNA expression between healthy controls and EOC patients. The diagnostic power of exosomal miRNAs was analyzed using receiver operating characteristic (ROC) curves. Missing data were handled by pairwise deletion. A *p* < 0.05 value was considered statistically significant with two-sided.

## Results

### Baseline clinical characteristics of the study population

This study included two datasets (Fig. S1). One was for smRNA sequencing comprising six EOC patients and four healthy controls. For the validation dataset, additional 17 healthy controls and 28 EOC patients were recruited (Table 1). The baseline clinical characteristics of the six EOC patients, including age, pathological type, breast cancer gene (BRCA) mutation status, and Federation of Gynecology and Obstetrics (FIGO, 2009) stage for smRNA sequencing are shown (Table 1). The median age of all EOC patients (*n* = 34) and healthy controls (*n* = 21) was 56 and 53 years, respectively. No significant differences in ages were recorded in both two datasets.

**Table 1:** Clinical characteristics of the 34 pre-operative plasma EOC patients and 21 healthy controls in discovery set and validation set

Data set	No.	Age (Year)	Pathological diagnosis	Stage	Grade	Pre-operative CA125(U/mL)	BRCA mutation status	Concurrent cancer
Discovery set	EOC1	65	EOC	IVB	NA^a^	NA	BRCA-	–
	EOC2	43	EOC	IIIC	NA	NA	BRCA-	–
	EOC3	67	EOC	IIIC	NA	NA	BRCA+	–
	EOC4	45	EOC	IIIC	NA	NA	BRCA-	–
	EOC5	57	EOC	IVB	NA	NA	BRCA+	–
	EOC6	55	EOC	IIIC	NA	NA	BRCA+	Breast cancer
	Ctrl1	52	Healthy control	–	–	–	NA	–
	Ctrl2	62	Healthy control	–	–	–	NA	–
	Ctrl3	48	Healthy control	–	–	–	NA	–
	Ctrl4	50	Healthy control	–	–	–	NA	–
Validation set	C1	56	EOC	IIIC	1	49.1	BRCA-	–
	C2	44	EOC	IIIC	3	6354	BRCA-	–
	C3	53	EOC	IIIC2	3	1648	BRCA-	–
	C4	56	EOC	IIIC	1	474.8	BRCA-	–
	C5	70	EOC	IIC	2	2221	BRCA-	–
	C6	60	EOC	IIIC	3	977.4	BRCA+	–
	C7	51	EOC	IIIC	3	93	BRCA-	–
	C8	54	EOC	NA	NA	12.9	NA	–
	C9	51	EOC	IIIC	3	1359	BRCA+	–
	C10	35	EOC	IIIA1	NA	253.9	BRCA-	–
	C11	61	EOC	IV	3	880.4	BRCA+	–
	C12	49	EOC	IIA	3	107	BRCA+	–
	C13	50	EOC	IIIB	3	690.7	NA	–
	C14	66	EOC	IIIC	NA	19.1	NA	–
	C15	43	EOC	IIIC	3	31.7	NA	–
	C16	52	EOC	IIIC	NA	63.9	NA	–
	C17	61	EOC	IIIC	NA	20,388.1	NA	–
	C18	49	EOC	IIIC	NA	923.9	NA	–
	C19	74	EOC	IV	NA	1311.1	BRCA+	–
	C20	51	EOC	IV	3	876.8	NA	–
	C21	61	EOC	IIIC	3	278	BRCA+	–
	C22	56	EOC	IIIC	3	3862	BRCA-	–
	C23	56	EOC	IC	3	191.2	BRCA-	–
	C24	46	EOC	IC	3	57.9	NA	–
	C25	65	EOC	IIC	3	128.4	BRCA+	–
	C26	64	EOC	IC	2	145.5	BRCA+	–
	C27	64	EOC	IV	NA	876.8	BRCA-	–
	C28	74	EOC	IIIC	NA	10.6	NA	–
	H1	42	Healthy control	–	–	–	–	–
	H2	59	Healthy control	–	–	–	–	–
	H3	46	Healthy control	–	–	–	–	–
	H4	53	Healthy control	–	–	–	–	–
	H5	54	Healthy control	–	–	–	–	–
	H6	55	Healthy control	–	–	–	–	–
	H7	41	Healthy control	–	–	–	–	–
	H8	53	Healthy control	–	–	–	–	–
	H9	45	Healthy control	–	–	–	–	–
	H10	56	Healthy control	–	–	–	–	–
	H11	37	Healthy control	–	–	–	–	–
	H12	60	Healthy control	–	–	–	–	–
	H13	58	Healthy control	–	–	–	–	–
	H15	34	Healthy control	–	–	–	–	–
	H16	54	Healthy control	–	–	–	–	–
	H18	55	Healthy control	–	–	–	–	–
	H20	43	Healthy control	–	–	–	–	–

^a^NA: not available, EOC/C: epithelial ovarian cancer, Ctrl/H: Healthy control, +/−: positive/ negative

### EV morphology using TEM

**Fig. 1:**
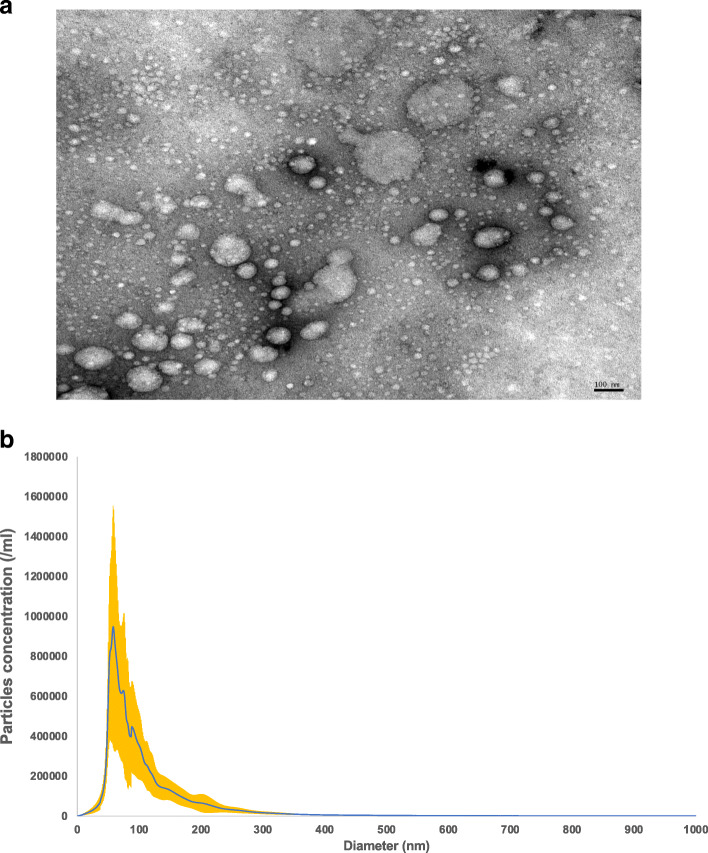
Identification of exosomes from plasma. **a** The black arrows indicate typical exosomes with size and morphology confirmed by TEM. Images were taken at 105,000 × magnification (scale bar = 100 nm). **b** Exosome particles from plasma were analyzed by NTA

To determine whether EVs were present in plasma extracts, TEM was used to investigate particle size and morphology. In Fig. 1a, black arrows indicated typical EV sizes, ranging from 30 to 150 nm in diameter, with characteristic round shapes. Size and particle distribution plots of plasma (Fig. 1b) indicated average diameters of 106.80 nm ± 30.84 nm in the typical exosome size range [49].

### Exosomal smRNA sequencing data quality control and composition changes

**Fig. 2:**
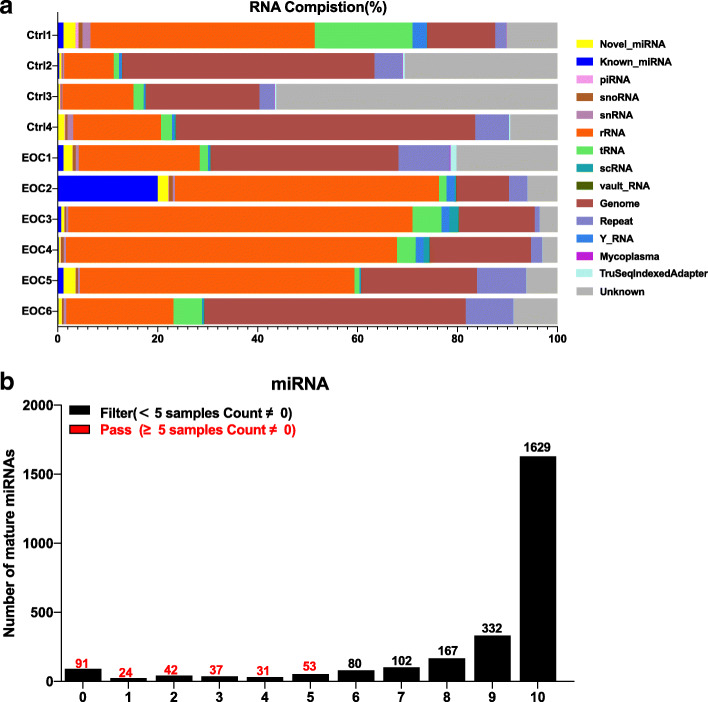
SmRNA sequence profiling and composition after data quality checks. Profiles of exosomal miRNAs after data quality checks. **a** smRNA composition of each sample, which means the ratio of smRNA class type. **b** Low expressed miRNAs were filtered by miRNA profiling. All transcript counts were filtered with more than zero in at least 50% (*n* ≥ 5) of all samples while miRNAs with zero counts in at least 50% samples are excluded

After library construction and sequencing in 10 samples, we obtained approximately 2.0–4.6 Giga base pairs (Gb) million raw bases in samples (Supplementary Table S1). An average 49,305,949 and 57,834,221 reads were produced in healthy controls and EOC patients, respectively. The GC content (%) was 21.57–44.87%, and the Q30 score which is the percentage of bases that have a Q-score above or equal to 30 was 79.88–90.18%. In Fig. S2, the x-axis shows the number of cycles and the y-axis shows the phred quality score. A phred quality score of 20 indicated 99% accuracy, and reads scoring > 20 indicated good quality. After the quantification of mature miRNA abundance, Supplementary Table S2 shows the number of mapped reads to the miRbase precursor, and the number of known mature miRNAs with read counts > 1 for each sample. To exosomal smRNA composition, we conducted NGS followed by mapping to each smRNA reference database. We identified exosomal smRNAs, including miRNAs, ribosomal RNAs (rRNAs), Piwi-interacting RNA (piRNA), and transfer RNAs (tRNAs). Nearly half (48%) on average of exosomal smRNAs were rRNAs. Figure 2a represents the smRNA composition of each sample, which means the ratio of smRNA class type classified from processed reads. A cumulative analysis of smRNA fractions was not significantly different between EOC patients and healthy control groups. If more than five samples that the read count value was 0, the expression was considered low and was excluded for further analysis. Therefore, from 2588 mature miRNAs, 2310 were excluded and 278 (Fig. 2b, red bars) were subject to further statistical analyses.

### Exosomal miRNA profiles in EOC patients

**Fig. 3:**
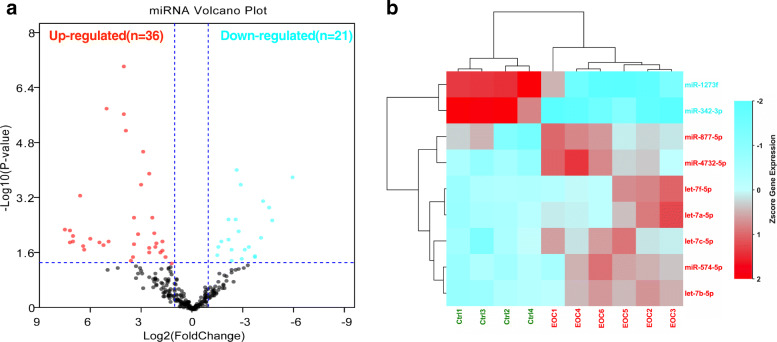
Differential expression and miRNA profiling of plasma derived exosomes from six ovarian cancer patients compared to four healthy controls. **a** Volcano plot depicting differential expression results for miRNAs in ovarian tumor samples when compared with healthy controls. Threshold:|Log_2_fold change | ≥ 1, *p* ≤ 0.05. **b** Heat-maps for differentially expressed miRNAs with a cut-off less than |Log_2_fold change | ≥ 1, p ≤ 0.05, FDR ≤ 0.05

After RNA sequencing, we identified 36 up-regulated and 21 down-regulated miRNAs in EOC patients when compared with healthy controls (Fig. 3a, Table 2). Two down-regulated miRNAs (miR-1273f and miR-342-3p) and seven up-regulated clustered miRNAs (miR-4732-5p, miR-877-5p, miR-574-3p, let-7a-5p, let-7b-5p, let-7c-5p, and let-7f-5p) was significantly noticed with a cutoff under |Log_2_ fold change | ≥ 1 and false discovery rate (FDR) ≤ 0.05 in six EOC patients (Fig. 3b). To validate the significant different expressed miRNAs detected by NGS, we selected miR-4732-5p (Log_2_ FC: 9.332, *p* = 0.01) which was highly up-regulated in EOC patients when compared with healthy controls for further study.

**Table 2:** Significant hits for exosomal miRNAs with their respective FC and FDR values

ID	logFC	PValue	FDR
**hsa-let-7b-5p**	**4.09248807**	**8.976E-08**	**2.4953E-05**
**hsa-miR-877-5p**	**5.15047208**	**1.4938E-06**	**0.00041378**
**hsa-let-7c-5p**	**4.12310127**	**2.165E-06**	**0.00059753**
**hsa-miR-574-5p**	**3.99226218**	**6.5504E-06**	**0.00180136**
**hsa-let-7a-5p**	**2.98567816**	**2.6637E-05**	**0.00729865**
**hsa-miR-4732-5p**	**9.33199983**	**3.718E-05**	**0.01015003**
**hsa-miR-342-3p**	**−2.5797631**	**8.9044E-05**	**0.02421989**
**hsa-let-7f-5p**	**2.61827187**	**0.00011794**	**0.03196047**
**hsa-miR-1273f**	**−5.8200372**	**0.00014525**	**0.03921859**
hsa-miR-92a-3p	3.10125041	0.00023975	0.06449215
hsa-miR-150-5p	−2.7995421	0.00024432	0.06547649
hsa-miR-1255b-5p	6.66611983	0.00050984	0.13612815
hsa-miR-619-5p	−4.1141977	0.00075219	0.2000831
hsa-miR-495-3p	−4.4429118	0.00113155	0.29986024
hsa-miR-505-5p	3.52456851	0.00216477	0.571499
hsa-miR-98-5p	2.43323632	0.00223196	0.58700634
hsa-miR-424-5p	−2.4668698	0.00250382	0.65600045
hsa-miR-335-5p	−2.0926881	0.00257234	0.67138077
hsa-miR-6126	−4.6666622	0.00269239	0.70002085
hsa-let-7e-5p	1.82022536	0.02001495	1
hsa-miR-107	2.5929338	0.01677811	1
hsa-miR-1246	2.31449036	0.00649523	1
hsa-miR-125a-5p	−2.6745151	0.00548481	1
hsa-miR-125b-5p	−2.8620099	0.03421185	1
hsa-miR-126-3p	−1.4837808	0.01628872	1
hsa-miR-142-3p	−2.0450917	0.00961864	1
hsa-miR-151a-3p	1.97746261	0.02258333	1
hsa-miR-183-5p	3.25504686	0.00677286	1
hsa-miR-1908-5p	5.52757268	0.0115459	1
hsa-miR-196b-5p	3.59633779	0.03062987	1
hsa-miR-197-5p	7.31202333	0.01196864	1
hsa-miR-19a-3p	−1.6797336	0.01082544	1
hsa-miR-19b-3p	−1.3927602	0.02750629	1
hsa-miR-206	7.08928709	0.0111442	1
hsa-miR-223-3p	1.31742496	0.04926554	1
hsa-miR-25-3p	2.19360414	0.01273148	1
hsa-miR-27a-3p	−2.2603729	0.01907464	1
hsa-miR-320d	3.67001804	0.03908426	1
hsa-miR-3605-5p	5.01799806	0.01105679	1
hsa-miR-361-3p	−2.2359632	0.04025571	1
hsa-miR-361-5p	−3.2663352	0.01573867	1
hsa-miR-376c-3p	−3.0264719	0.0193028	1
hsa-miR-423-5p	1.83075454	0.01118424	1
hsa-miR-425-5p	2.63170485	0.02224773	1
hsa-miR-4446-3p	6.51702353	0.01502838	1
hsa-miR-4644	5.29392303	0.01438286	1
hsa-miR-483-3p	−4.0174211	0.00856955	1
hsa-miR-483-5p	3.4825009	0.01339968	1
hsa-miR-486-5p	1.63381156	0.03029494	1
hsa-miR-5096	− 3.6296818	0.02877727	1
hsa-miR-5187-5p	7.27793006	0.00519647	1
hsa-miR-628-5p	7.57777486	0.00506898	1
hsa-miR-642a-3p	6.06929969	0.00914869	1
hsa-miR-6734-5p	7.10300091	0.00742654	1
hsa-miR-6873-3p	−3.6005875	0.03092883	1
hsa-miR-92b-3p	2.25929664	0.01628188	1
hsa-miR-942-5p	6.44631142	0.01942818	1

### Plasma derived exosomal miR-4732-5p may be a promising circulating predictive biomarker in EOC

**Fig. 4:**
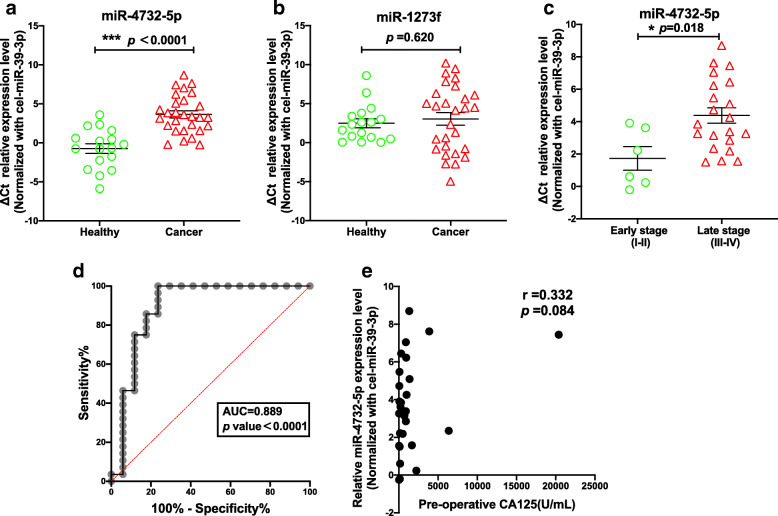
Plasma derived exosomal miR-4732-5p as a diagnostic biomarker. SYBER green quantitative RT-PCR indicated that miR-4732-5p (**a**) was significantly up-regulated in ovarian cancer patient plasma derived exosomes when compared with healthy controls (*p* = 0.003),while there was no significant changed for miR-1273f (**b**). **c** Plasma derived exosomal miR-4732-5p was significantly up-regulated in late-stage ovarian cancer patient when compared with early-stage group (*p* = 0.016). **d** ROC curve analysis indicated that the AUC of miR-4732-5p between ovarian cancer patients and healthy controls was 0.889 (p<0.0001). **e** The correlation between CA-125 level and miR-4732-5p

To validate predicted miRNA biomarkers, plasma derived exosomes from 28 EOC patients were further analyzed. When compared with the healthy group, miR-4372-5p was significantly up-regulated in EOC patients (Fig. 4a, p <0.0001) without significant difference for miR-1273f (Fig. 4b, p =0.620). Moreover, miR-4372-5p was significantly increased in 19 late-stage EOC patients compared to six early-stage patients (Fig. 4c, p =0.018), showing a promising capacity for use as a diagnostic marker of cancer stage. When normalized to cel-miR-39, exosomal miR-4732-5p had an area under the curve (AUC) of 0.889 (Fig. 4d, p <0.0001), with 85.7% sensitivity and 82.4% specificity. Additionally, we didn’t observe any correlation between CA-125 levels and miR-4732-5p expression which may improve clinical sensitivity for EOC (r = 0.332, *p* = 0.084). There is no correlation between the level of exosomal miR-4732-5p and BRCA mutation status or pathological grading (Fig. S3).

## Discussion

Novel sensitive, specific and stable circulating miRNAs are promising non-invasive biomarkers for the timely and effective diagnosis and treatment of ovarian cancer. In our study, we initially conducted smRNA sequencing to examine circulating exosomal miRNA profiles in patients with EOC in the discovery set. We identified 36 up-regulated and 21 down-regulated EOC-specific miRNAs whose levels were deregulated in circulating exosomes. The most up-regulated miRNAs were, miR-4732-5p, miR-877-5p, miR-574-3p, let-7a-5p, let-7b-5p, let-7c-5p, and let-7f-5p, and the most down-regulated miRNAs were miR-1273f, and miR-342-3p. However, these miRNAs did not overlap with the miRNAs identified from previous studies [50, 51].

Some previous pilot studies which were focused either on miRNA signatures by microarrays, or on miRNA profiling by NGS technical tools showed variable results. For example, in Iorio’s study [52], the most over-expressed miRNAs were miR-200a, miR-141, miR-200c, and miR-200b, while the most under-expressed miRNAs were miR-199a, miR-140, miR-145, and miR-125b in 69 snap-frozen malignant ovarian tissues compared with 15 normal ovary tissues sections by microarray analysis. In most high-grade serous ovarian carcinoma, miR-182 was found to be significantly over-expressed and its oncogenic role was also validated in tumor transformation, invasiveness and metastasis in vitro and vivo [53]. Another study reported that miR-508-3p, miR-509-3p, miR-509-3-5p, and miR-514a-3p were remarkably downregulated in recurrent ovarian clear cell cancer tissues when compared with those in paired primary cancer tissues [54]. Overexpression of miR-509-3p and miR-509-3-5p could reverse cisplatin resistance in vitro [54]. In a small cohort [55] including 20 serous ovarian carcinoma tissues and 8 benign uterine disease samples has identified 23 significant deregulated miRNAs such as miR-21, miR-125a, miR-125b, miR-100, miR-145, miR-16, and miR-99a by DNA microarray and Northern blot analyses. A large number of miRNA profilings of 487 ovarian cancers from the Cancer Genome Atlas (TCGA) database revealed several miRNAs in focally amplified and deleted genomic regions [56]. For instance, miR-31, a deleted miRNA could impede ovarian cancer cell proliferation. Besides that, let-7b was the most frequently deleted miRNA having both recurrent hemizygous genomic loss (86% of samples) and homozygous deletion (7.2%). In Taylor’s study [57], eight tumors derived from exosome microRNAs (miR-21, miR-141, miR-200a, miR-200c, miR-200b, miR-203, miR-205 and miR-214) could serve as diagnostic markers of ovarian cancer.

Of note, the sampling of studies mentioned above were all from cancer tissues, while a few studies [50, 51, 58] have focused on plasma exosomal miRNAs in EOC. For instance, miR-93, miR-145, and miR-200c showed significantly higher expression in an ovarian cancer group than of the benign group [58]. Specifically, miR-145 showed superior sensitivity (91.6%) which could be a promising biomarker for preoperative diagnosis of ovarian cancer. Recently, Maeda et al. [37] found that serum exosomal miR-34a was significantly elevated in early-stage ovarian cancer compared to late-stage group, as the similar results reported by Zhang et al. [59]. In 15 platinum-resistant patients, plasma derived of miR-181a, miR-1908, miR-21, miR-486 and miR-223 were differentially abundant compared to 15 platinum-sensitive plasma (*n* = 15) [60]. However, in our study, the exosomal miR-4732-5p was statistically elevated (3.6-fold) in EOC patients when compared with healthy women, with adequate high sensitivity and specificity to discriminate EOC from healthy women, indicating that miR-4732-5p may be a good diagnostic biomarker for EOC. The inconsistent results and specific patterns may be due to unavoidable co-purified circulating particles by different methods for EV-extraction and the inter-patient differences in the EV-associated miRNA profiles [60]. Interestingly, the relative expression of serum exosomal miR-4732-5p in our study was significantly higher in late-stage OC patients than in early-stage patients, which was consistent with the results reported in breast cancer. This data was consistent with the results reported in breast cancer, showing a potential ability of serum exosomal miR-4732-5p as a biomarker of late-stage diagnosis in different cancer [61]. However, the potential ability of serum exosomal miR-4732-5p as a biomarker for late-stage diagnosis in OC needs a further study in a larger cohort.

To our knowledge, few studies have focused on miR-4732-5p, with data indicating distinct and dual roles for miR-4732-5p in different cancers. For example, Fukumoto et al. [62] using array-based methods, observed that miR-4732-5p was highly expressed in hypopharyngeal squamous cell carcinoma tissues when compared with normal tissues. MiR-4732-5p played a major oncogenic role in breast cancer progression by targeting TSPAN13 [61], and potentially served as an unfavorable biomarker with miR-448, miR-486, miR-516, and miR-1911 in lung squamous cell carcinoma [63]. Up-regulated plasma based miR-4732-5p was also reported as a diagnostic and prognostic marker in pancreatic ductal adenocarcinoma patients when compared with healthy group [64]. Moreover, miR-4732-5 directly bound the 5′-untranslated regions (UTRs) of Wrap53 mRNA in breast cancer, and prohibited p53 mRNA binding [65], indicating a possible link between miR-4732-5p and the tumor suppression protein, p53 [65]. Up-regulated miR-4732-5p was closely associated with disease relapse after S-1 adjuvant chemotherapy treatment in gastric cancer [66]. Moreover, up-regulated miR-4732-5p expression signals (26-fold) were detected outside (supernatants) cancer cells in both colon and pancreatic cancer cells, indicating the putative activation of an export mechanism for tumor suppressor miRNAs [65]. In contrast, Zhang et al. [67] reported that miR-4732-5p was down-regulated in nipple discharge compared to benign intraductal papilloma patients, which is controversial with the oncogenic role of miR-4732-5p playing in lymph node metastasis (LNM)-positive breast cancer during tumor progression as previously reported [61]. When compared with normal adjacent tissues, miR-4732-5p was significantly down-regulated in breast cancer tissues in LNM-negative tissues, indicating for the same cancer, miR-4732-5p appeared to have dual roles in tumor initiation and progression.

It was reported that some miRNAs may have distinct and opposing roles at different stages or cancer origins [61, 67]. For instance, miR-145, which was identified as a suppressor gene marker in ovarian cancer tissue in previous studies [68], highly expressed in the serum exosomes of in their cancer patients in Kim’s study [58], revealing that miRNAs expression in serum exosomes does not always mirror that of the originating tumor tissue [58]. Moreover, miR-320 elevated expression was associated with a poor prognosis, migration and invasion and high risk of metastasis in EOC patients [69]. In contrast, another study revealed miR-320 down-regulation in EOC served as a tumor suppressor when compared with matched adjacent normal ovarian epithelium tissues; it suppressed cell proliferation and invasion by targeting mitogen-activated protein kinases (MAPK1) [70]. Research by Pan et al. [51] reported that miR-320 was preferentially packaged into exosomes in EOC patients, but it had no impact on cell proliferation and apoptosis in ovarian cancer cell lines. These contradictory studies suggest that the same miRNAs may have different roles and could functionally vary in terms of source and cancer stages. Therefore, more in vitro and in vivo studies must be conducted to address the role of miR-4732-5p in tumor cells and explore its function(s) during EOC progression in future.

Our study had some limitations. Firstly, follow-up data was missing, thus overall survival rates and progression-free survival rates were not analyzed. A larger cohort will be required to assess if exosomal miR-4732-5p may be utilized as a potential prognostic predictor of EOC. Secondly, it was previously reported that high levels of invasive ovarian cancer cells could release significantly more exosomes than low-invasive cells [71], showing the potential to increase cell invasion from premetastatic niches to prepare secondary sites for metastasis. However, discriminating EOC patients at early stages from advanced stages was not possible in our dataset due to a lack of pathological staging data. Moreover, compared to cancer patients, exosomes in healthy women blood were lower and mainly derive from platelets, erythrocytes, and endothelial cells [72]. Circulating exosome could also derive from different cells (like immune cells, mesenchymal stem or stromal cells), not only circulating tumor cells. We propose that mechanisms underlying miR4732–5p’s ability in EOC progression require further exploration.

## Conclusions

Plasma derived exosomal miR-4732-5p was significantly up-regulated EOC, and may serve as a potential biomarker for the diagnosis of this condition.

## Supplementary Information

**Additional file 1.** Materials and Methods: SmRNA library preparation and sequencing.

**Additional file 2: Table S1**. Raw data statistics.

**Additional file 3: Table S2**. Mapped reads to miRbase precursor.

**Additional file 4: Figure S1.** Study workflow.

**Additional file 5: Figure S2.** Quality control of the data.

**Additional file 6: Figure S3.** Correlation between the level of exosomal miR-4732-5p and BRCA mutation status or pathological grade.

## Data Availability

The data that support the findings of this study are available on request from the corresponding author.
