# Supplementary material for: Plasma-derived exosomal miR-4732-5p is a promising noninvasive diagnostic biomarker for epithelial ovarian cancer
Source: J Ovarian Res. 2021 Apr 28;14:59. doi: 10.1186/s13048-021-00814-z (PMC8082916; doi:10.1186/s13048-021-00814-z)
Supplement: Supplementary file 1 — Additional file 1. Materials and Methods: SmRNA library preparation and sequencing. [file 13048_2021_814_MOESM1_ESM.docx]

**Materials and Methods**

**SmRNA library preparation and sequencing**

The cDNAs were subjected to end-repair and poly-A addition and connected with sequencing adapters (Illumina, San Diego, CA). The suitable fragments were automatically purified by the BluePippin 2 % agarose gel cassette (Sage Science, Beverly, MA) and selected as templates for polymerase chain reaction (PCR) amplification. The final library sizes and qualities were assessed electrophoretically by an Agilent High Sensitivity DNA kit (Agilent Technologies, Santa Clara, CA), and the size of fragment was found to be between 350 - 450 bp. Compared to the manufacturer’s protocol, the fragmentation step was set to 4 min at 94 °C, hereafter the option to start from highly degraded RNA was followed. Library quality control was performed with the Fragment Analyzer high sense small fragment kit (Agilent Technologies, sizing range 50 - 1000 bp). Based on Qubit concentration measurements or KAPA qPCR, samples were pooled and loaded on the Illumina HiSeq 2500 sequencer (Illumina, San Diego, CA, USA).

RNA sequencing data were aligned to the human genome (GenBank accession no. hg19) and processed by Bowtie, HISAT2 and STAR using RSEM. The raw sequence reads are filtered based on quality (a low quality score (Q30) were discarded) with the adapter sequences are trimmed off by cutadapt (v.1.16). Both the processed trimmed reads and non-adapter reads are used to analyze long target (≧ 50bp). Trimmed reads were considered miRNA based on having 100 % identical and full-length miRNA sequences as compared with those in the miRBase database.
