# Supplementary material for: Plasma-derived exosomal miR-4732-5p is a promising noninvasive diagnostic biomarker for epithelial ovarian cancer
Source: J Ovarian Res. 2021 Apr 28;14:59. doi: 10.1186/s13048-021-00814-z (PMC8082916; doi:10.1186/s13048-021-00814-z)
Supplement: Supplementary file 2 — Additional file 2: Table S1. Raw data statistics. [file 13048_2021_814_MOESM2_ESM.docx]

**Table S1. Raw data stats**

| **Sample** | **Total read bases** | **Total reads** | **GC (%)** | **Q20 (%)** | **Q30 (%)** |
| --- | --- | --- | --- | --- | --- |
| Ctrl1 | 3,104,623,011 | 60,874,961 | 40.38 | 89.90 | 83.07 |
| Ctrl2 | 2,013,145,644 | 39,473,444 | 40.79 | 91.61 | 85.80 |
| Ctrl3 | 2,380,608,039 | 46,678,589 | 38.79 | 93.45 | 88.84 |
| Ctrl4 | 2,560,036,851 | 50,196,801 | 33.49 | 89.62 | 83.08 |
| EOC1 | 3,014,358,060 | 59,105,060 | 21.57 | 91.80 | 85.67 |
| EOC2 | 4,592,241,960 | 90,043,960 | 39.40 | 87.59 | 79.88 |
| EOC3 | 2,740,692,621 | 53,739,071 | 44.87 | 92.73 | 87.96 |
| EOC4 | 2,750,597,025 | 53,933,275 | 40.20 | 94.20 | 90.18 |
| EOC5 | 2,184,355,092 | 42,830,492 | 31.19 | 88.54 | 81.81 |
| EOC6 | 2,415,026,817 | 47,353,467 | 35.23 | 91.96 | 86.96 |

Total read bases (Total reads x Read length): Total number of bases sequenced; Total reads: Total number of reads; GC (%): GC content Q20 (%): Ratio of bases that have phred quality score greater than or equal to 20; Q30 (%): Ratio of bases that have phred quality score greater than or equal to 30
