# Supplementary material for: Plasma-derived exosomal miR-4732-5p is a promising noninvasive diagnostic biomarker for epithelial ovarian cancer
Source: J Ovarian Res. 2021 Apr 28;14:59. doi: 10.1186/s13048-021-00814-z (PMC8082916; doi:10.1186/s13048-021-00814-z)
Supplement: Supplementary file 3 — Additional file 3: Table S2. Mapped reads to miRbase precursor. [file 13048_2021_814_MOESM3_ESM.docx]

**Table S2. Mapped reads to miRbase precursor**

| **Sample** | **Processed reads** | **Mapped reads** | **Known miRNA in sample** | **Known miRNA**  **in Species**  **(miRbase v21)** |
| --- | --- | --- | --- | --- |
| Ctrl1 | 13,758,559 | 300,161 (2.18%) | 585 | 2,588 |
| Ctrl2 | 20,697,524 | 71,129 (0.34%) | 199 | 2,588 |
| Ctrl3 | 26,138,255 | 48,720 (0.19%) | 196 | 2,588 |
| Ctrl4 | 10,516,171 | 17,492 (0.17%) | 175 | 2,588 |
| EOC1 | 9,329,006 | 136,890 (1.47%) | 243 | 2,588 |
| EOC2 | 12,738,602 | 5,359,240(42.07%) | 777 | 2,588 |
| EOC3 | 10,143,201 | 224,154 (2.21%) | 404 | 2,588 |
| EOC4 | 10,825,614 | 68,269 (0.63%) | 296 | 2,588 |
| EOC5 | 6,028,896 | 154,612 (2.56%) | 344 | 2,588 |
| EOC6 | 23,082,675 | 48,945 (0.21%) | 245 | 2,588 |
