# Supplementary material for: Plasma-derived exosomal miR-4732-5p is a promising noninvasive diagnostic biomarker for epithelial ovarian cancer
Source: J Ovarian Res. 2021 Apr 28;14:59. doi: 10.1186/s13048-021-00814-z (PMC8082916; doi:10.1186/s13048-021-00814-z)
Supplement: Supplementary file 4 — Additional file 4: Figure S1. Study workflow. [file 13048_2021_814_MOESM4_ESM.pdf]

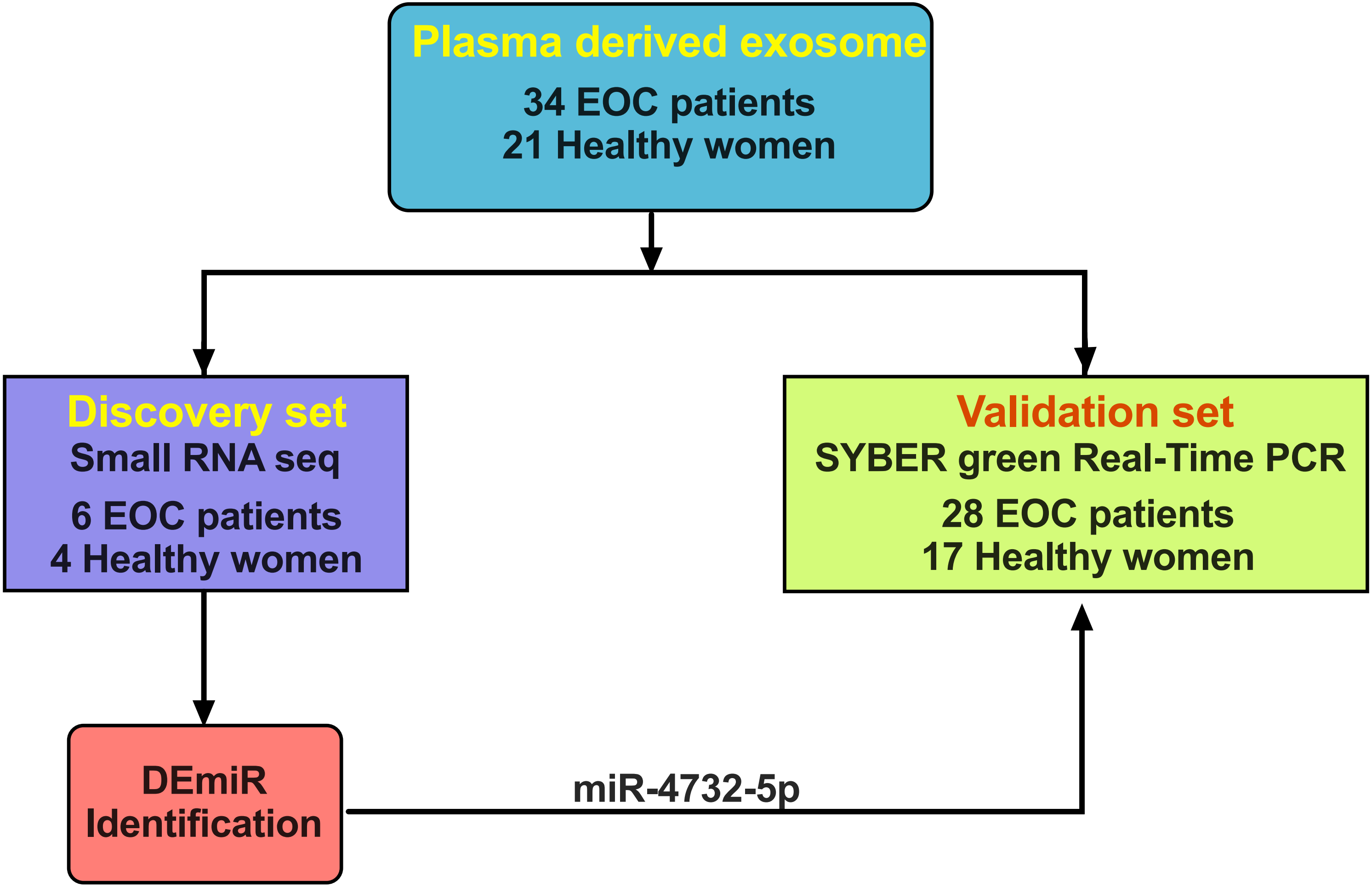

Fig. S1 Study workflow. The schematic pipeline for plasma derived exosomal EOC miRNA biomarker identification. EOC: Epithelial Ovarian Cancer; DEmiRs: Differentially expressed miRNAs.
