# Supplementary figures and images for: Plasma-derived exosomal miR-4732-5p is a promising noninvasive diagnostic biomarker for epithelial ovarian cancer
Source: J Ovarian Res. 2021 Apr 28;14:59. doi: 10.1186/s13048-021-00814-z (PMC8082916; doi:10.1186/s13048-021-00814-z)

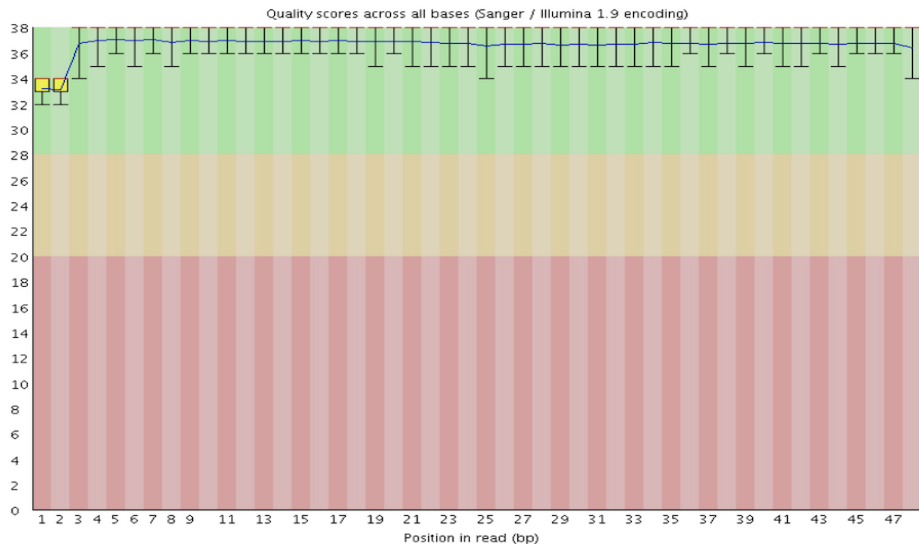

Average base quality at each cycle of one sample, EOC6 (read 1).

Supplement: Supplementary file 5 — Additional file 5: Figure S2. Quality control of the data. [file 13048_2021_814_MOESM5_ESM.pdf]
