# Supplementary material for: Plasma-derived exosomal miR-4732-5p is a promising noninvasive diagnostic biomarker for epithelial ovarian cancer
Source: J Ovarian Res. 2021 Apr 28;14:59. doi: 10.1186/s13048-021-00814-z (PMC8082916; doi:10.1186/s13048-021-00814-z)
Supplement: Supplementary file 6 — Additional file 6: Figure S3. Correlation between the level of exosomal miR-4732-5p and BRCA mutation status or pathological grade. [file 13048_2021_814_MOESM6_ESM.pdf]

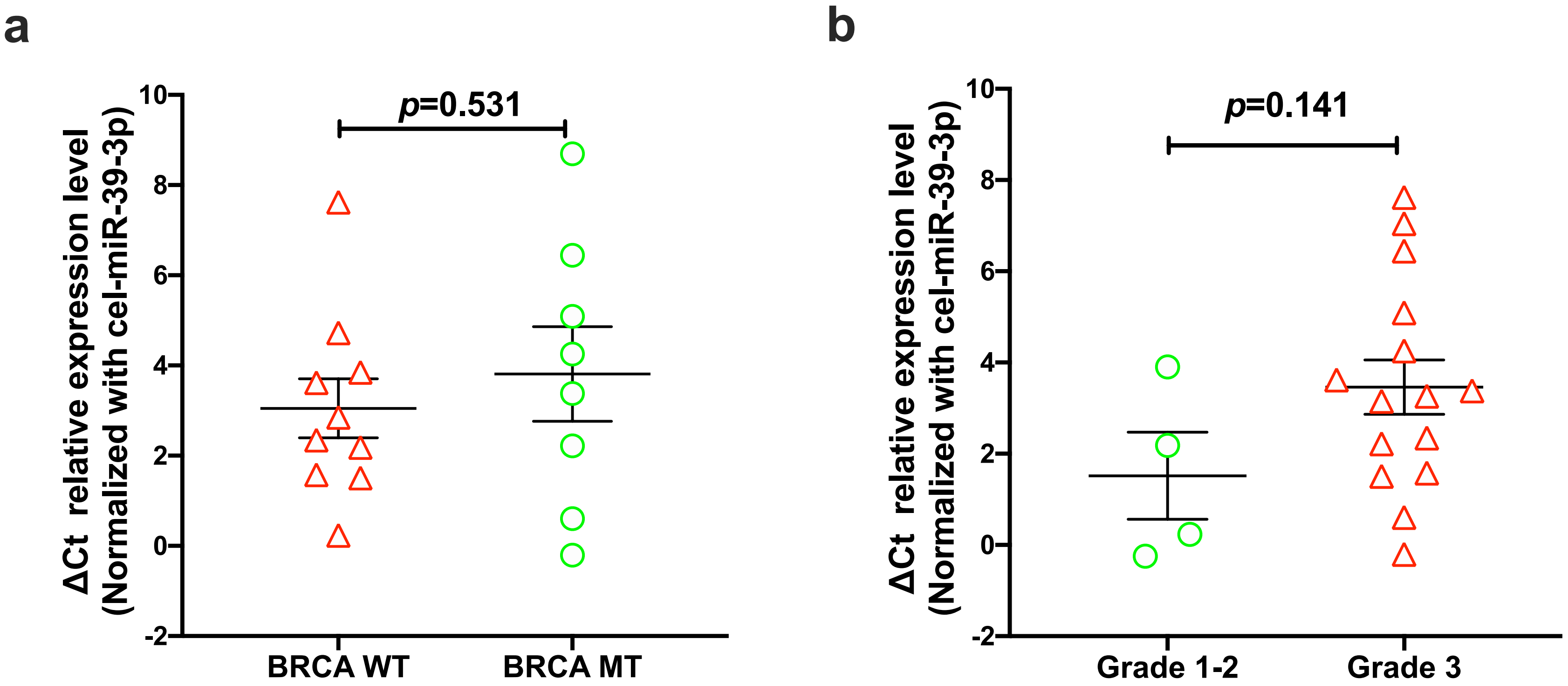

SYBER green quantitative RT-PCR indicated that there is no corelationship between exosomal miR-4732-5p expression level and BRCA mutation status (**a**) , pathological grading (**b**).
